# Variation in Protein Intake Induces Variation in Spider Silk Expression

**DOI:** 10.1371/journal.pone.0031626

**Published:** 2012-02-20

**Authors:** Sean J. Blamires, Chun-Lin Wu, I-Min Tso

**Affiliations:** 1 Department of Life Science, Tunghai University, Taichung, Taiwan; 2 Center for Measurement Standards, Industrial Technology Research Institute, Hsinchu, Taiwan; 3 Department of Life Science, National Chung-Hsing University, Taichung, Taiwan; Aarhus University, Denmark

## Abstract

**Background:**

It is energetically expensive to synthesize certain amino acids. The proteins (spidroins) of spider major ampullate (MA) silk, MaSp1 and MaSp2, differ in amino acid composition. Glutamine and proline are prevalent in MaSp2 and are expensive to synthesize. Since most orb web spiders express high proline silk they might preferentially attain the amino acids needed for silk from food and shift toward expressing more MaSp1 in their MA silk when starved.

**Methodology/Principal Findings:**

We fed three spiders; *Argiope aetherea*, *Cyrtophora moluccensis* and *Leucauge blanda*, high protein, low protein or no protein solutions. *A. aetherea* and *L. blanda* MA silks are high in proline, while *C. moluccesnsis* MA silks are low in proline. After 10 days of feeding we determined the amino acid compositions and mechanical properties of each species' MA silk and compared them between species and treatments with pre-treatment samples, accounting for ancestry. We found that the proline and glutamine of *A. aetherea* and *L. blanda* silks were affected by protein intake; significantly decreasing under the low and no protein intake treatments. Glutmaine composition in *C. moluccensis* silk was likewise affected by protein intake. However, the composition of proline in their MA silk was not significantly affected by protein intake.

**Conclusions:**

Our results suggest that protein limitation induces a shift toward different silk proteins with lower glutamine and/or proline content. Contradictions to the MaSp model lie in the findings that *C. moluccensis* MA silks did not experience a significant reduction in proline and *A. aetherea* did not experience a significant reduction in serine on low/no protein. The mechanical properties of the silks could not be explained by a MaSp1 expressional shift. Factors other than MaSp expression, such as the expression of spidroin-like orthologues, may impact on silk amino acid composition and spinning and glandular processes may impact mechanics.

## Introduction

Protein is integral for organismal function. Organisms exposed to protein limited environments hence must carefully partition ingested protein between somatic and metabolic requirements [1], [2]. Animals that synthesize and secrete proteinaceous materials potentially face further protein stresses 3–5. These may be partially alleviated by the metabolic synthesis of the amino acids required to build the materials [3], [6]. Nonetheless synthesizing amino acids comes at metabolic costs, which vary depending on the structural complexity of the amino acid and the metabolic phase it is derived from [2]. Primarily, amino acids that are derived from pre-citric acid cycle metabolites such as glucose 6-phosphate (e.g. histidine), 3-phosphoglycerate (e.g. serine and glycine) and pyruvate (e.g. alanine, leucine) are synthesized at a lower energetic cost than those derived from citric acid cycle metabolites such as oxaloacetate (e.g. asparagine, methionine) and α-ketoglutarate (e.g. glutamine, proline) [3], [6]–[9]. Further, certain amino acids, the so-called “essential amino acids” cannot be derived metabolically [6]. As amino acid biosynthesis is associated with the sacrifice of energy and retention of nitrogenous toxins [2], [6], [7], uptake from food is the principal method of obtaining the requisite amino acids for protein synthesis by most animals.

The silks of silk worms and spiders are examples of secreted proteinaceous materials [10], [11]. Researchers are particularly interested in understanding the metabolic costs and synthetic pathways of spider silk because its combined properties of high strength and extensibility and ability to be synthesized in a non-toxic environment render it desirable to commercially develop [3], [9]–[14]. Nevertheless, how nutrient intake, especially protein, influences silk synthesis and expression, and the performance consequences of any variations in silk expression are still poorly understood in spiders.

Web building spiders may produce up to seven different types of silk [11], [15]. Nonetheless, research to date has focused principally on major ampullate (MA) silk as this is the silk that has the most impressive mechanical properties. MA silk has been described to consist of two proteins; major ampullate spidroin 1 and 2, or MaSp1 and MaSp2 [16]–[18]. MaSp1 consists of alanine (Poly-A) and glycine (GGX-) repetitive motifs [8]–[10]. MaSp2 contains, in addition to these alanine and glycine motifs, a proline-containing motif (–GPG). It thus may be possible to estimate, depending on the spider, the relative quantity of MaSp1 and MaSp2 in a sample of MA silk based on the relative amounts of alanine (which may range from ∼15–35% depending on methods), glycine (ranging from ∼30–45% depending on methods) and proline (ranging from ∼0% in silks entirely composed of MaSp1 to ∼15% in silks entirely composed of MaSp2, depending on methods) [19]–[23]. Accordingly, most orb-web spiders, with the exception of some species of *Nephila* [20]–[23], *Cyrtophora* [20], [21] and *Latrodectus* [20], [24], appear to have MA silks that principally comprise of MaSp2. The principal reason for the predominance of MaSp2 expression in orb web spider MA silk is probably associated with the predicted β-spiral molecular arrangement of the MaSp2 spidroin as it endows the silk with a combination of strength and extensibility [16], [20]; properties essential for the prey impact absorption function of spider orb webs [11], [12]. Having repeating units consisting of proline and glutamine, the MaSp2 spidroin seems to be more energetically expensive to synthesize metabolically [4], [8], [9]. For this reason it was predicted that the golden orb web spider, *Nephila clavipes*, expresses less MaSp2 in its MA silk when under starvation stress [8], [25].

The two-spidroin (MaSp) model for spider MA silk was derived from detailed studies of the underlying genetics and the chemical and physical properties under supercontraction of the MA silk of a model spider, *Nephila clavipes* [8], [19], [26]–[29]. Nonetheless, as more spider silks are examined, contradictions to the model have arisen, bringing its universal applicability into question. For example, individuals of the giant wood spider, *Nephila pilipes* found in different regions of Taiwan, and/or that feed on different prey, have variations in the amino acids expressed in their MA silk but the variations are associated with changes in alanine and glycine but not proline [30]–[32]. Such variations, accordingly, cannot be explained by shifts in MaSp expression [32]. Additionally, the riverine orb spider of Madagascar, *Caerostris darwinii*, exhibits silk with such extreme extensibility and toughness that it cannot be explained by the expression of a combination of MaSp1 and MaSp2 [33]. One potential explanations for these contradictions is the possibility that spiders express multiple, unidentified, orthologues of MaSp1 or MaSp2, as described for *Araneus diadematus* [17], [34], [35]. Additionally, factors such as the physiological and biochemical state of the spider and spinning processes act on the molecular alignment of the proteins and consequently alter the mechanical properties of the dry silk irrespective of the influence of MaSp expression [25], [28], [32], [36]–[38].

Here we expand on research suggesting that the amino acid composition and mechanical properties of spider silks are altered in accordance with the diet of the spider [8], [25], [30]–[32], [39], [40]. We recently suggested that the nutritive value of prey can induce differential expression of spider MA silk but we were unsuccessful at completely decoupling nutrients from other influential prey parameters, such as the size and handling characteristics of the different prey [32]. This study thus investigates the specific role of protein intake as an inducer of variation in spider MA silk. We assume that certain amino acids (e.g. proline and glutamine) are required by spiders for silk synthesis and silk functionality but are expensive to attain through the diversion of metabolites and protein uptake via food or re-ingestion of the web, which may contribute over 90% of the protein required for some silks [4], [8], [9], is principally relied upon for their acquisition. We also test whether the effects of protein uptake varies in different spiders, as different spiders may produce MA silks of varying amino acid composition [41], which may, if the MaSp model holds, reflect their predicted MaSp expression [16], [21], [22], [26], [27].

We compared the MA silk expression (i.e. amino acid composition and mechanical properties) of three orb-web spiders: *Argiope aetherea*, *Leucauge blanda* and *Cyrtophora moluccensis* under three protein intake regimes: high, low or no protein intake. While the relative genetic inputs into the silks of these species are unknown, species of the former two genera have been reported to exhibit high proline (∼9–12%), hence most likely MaSp2 predominant, MA silks [20], [21], [41], [42]. Species from the latter genus exhibit low proline (∼1–2%), hence most likely MaSp1 predominant, MA silks [20], [21]. Nonetheless, phylogenetically *Argiope aetherea* and *Cyrtophora moluccensis* are more closely related with *Leucauge blanda* distantly related to the former two species [43].

We tested two predictions: (1) that proline and glutamine content in the MA silks of *A. aetherea* and *L. blanda* will decrease when feeding on low or no protein compared to when feeding on high protein. However, the proline and glutamine composition in *C. moluccensis* MA silks will not be as manifestly influenced by protein intake, indicative of a shift in expression away from MaSp2 expression in *A. aetherea* and *L. blanda*. Such a result would corroborate the premise that glutamine and proline uptake from food or re-ingestion of webs is primarily relied upon to deliver these amino acids for silk synthesis [8], and this requirement is greater in orb web spiders that produce MaSp2 predominant silks. Alternatively, (2) silk expression responses to protein intake may be explained by phylogeny [44], [45]. In this case, we would expect that the glutamine and proline compositions of *A. aetherea* and *C. moluccensis* to exhibit similar shifts, which should differ from those of *L. blanda*. We assumed any findings other than those we have predicted to indicate that factors other than protein intake or phylogeny act as the bases for shifts in MA silk expression with diet. The relationship between amino acid composition and mechanical properties of the MA silks of these species across the three feeding treatments were used to determine whether protein intake confers any effects on MA silk mechanical performance and whether the MaSp model is able to explain the changes ascertained.

## Methods

### Ethics statement

Ethic clearance was not required to perform this research. Capture permits were not required under Taiwan law as all collections were made outside of protected areas.

### Spider collection and pre-treatments

We collected adult female *Argiope aetherea*, *Cyrtophora moluccensis* and *Leucauge blanda* (*n* = 21 each) from Taichung, Nantou and Taitung counties in Taiwan on separate trips in July and August 2010. Spiders were weighed in the field upon capture before being returned to the laboratory at Tunghai University, Taichung. To minimize the amino acid composition variations in the “pre-treatment” silks as a result of diet and environmental factors experienced in the field, all spiders were acclimated by being placed in 0.5 *l* plastic cups with cotton mesh lids from the time of capture and fed one *Drosophila melanogaster* daily over 7 days. The following experiment was done for each species and ran for 10 days.

### Experiment

Each spider was randomly assigned to either a: (*i*) high protein (HP), (*ii*) low protein (LP) or (*iii*) no protein (NP) feeding treatment (*n* = 7 per treatment). The HP solution comprised of 20 m*l* chicken egg albumin, a saturated (∼0.16 g m*l* ^−1^ at 25°C) glycoprotein, carbohydrate, phosphate and trace element solution, mixed in 30 m*l* 0.2 g m*l* ^−1^ sucrose solution. The LP solution was a mixture of 10 m*l* chicken egg albumin in 40 m*l* 0.2 g m*l* ^−1^ sucrose solution. The HP and LP solutions were comprised principally of the amino acids lysine, glycine, cytesine, asparagine, glutamine and proline, as these constitute >85% of the amino acids of chicken albumin [46]. The NP solution comprised of just the 0.2 g m*l* ^−1^ sucrose solution. Chicken egg albumin was chosen because it is commercially available, has a high biological value, i.e. its protein, energy and minerals are readily assimilated by most animals including spiders [39], and it is saturated at similar concentrations as sucrose.

The solutions were analyzed in a single-channel CHN analyzer (Malvern Instruments, Malvern, U.K.) at the Department of Food Sciences, Tunghai University. The HP solutions had, by dry weight, 55.5% protein and 28.7% carbohydrate (approximately 2∶1 protein: carbohydrate ratio) and the LP solution had 24.6% protein: 59.5% carbohydrate (approximately 1: 2 protein: carbohydrate ratio). These values are approximately representative of extreme dry weight protein: carbohydrate ratios found naturally in insects [47]. As protein and carbohydrates contain approximately similar energy densities (4 kJg^−1^) using solutions differing only in protein: carbohydrate ratio meant the total energy across treatments was similar, assuming that the energy derived from albumin protein is as readily metabolized by spiders as that derived from sucrose.

We soaked 75 mm long cotton swabs in 1 m*l* of solution for approximately 5 min. We weighed each swab before and after soaking to ensure ∼0.1 g of food was absorbed. The soaked swabs were inserted into a fine (∼1 mm) slit, cut using a Stanley knife, into the centre of each cup's mesh on its lid. The swabs were pushed approximately 75% of their length into the cup to ensure they hung rigidly in the middle of the cup. The swabs were removed and re-weighed at 0800 h every day before being replaced. We determined the amount of food consumed per unit weight of spider, accounting for evaporation, measured as the weight change of the swab less that of a swab soaked with ∼0.1 g of the same solution left in a cup for 1 day without being fed from. We found no significant difference across species or treatments (2-factor, species×treatment, Kruskall-Wallis statistic = 7.63; *p* = 0.37). One NP fed *L. blanda* died during the course of the experiment so it was excluded from analyses. We did not feed the spiders fixed prey reared on manipulated media [48], [49] because unaccounted inter-individual variations in prey protein content and consequent behaviours alters spider feeding behaviour [50]. Moreover, we wanted to prevent spiders from building webs to circumvent any confounding influence that web building has on silk expression. We did not deliver the solutions by pipette [39] because *A. aetherea* and *L. blanda* consistently retreat from approaching instruments.

### Silk collection, amino acid assays and tensile testing

We collected silks: (*i*) after 7 days acclimation on a standardized feeding regime (see ‘Spider collection and pre-treatments’) to minimize the amino acid composition variations (pre-treatment samples), and (*ii*) after 10 days of the feeding treatment (post-treatment samples). Before collecting the silks all spiders were anaesthetized using CO_2_ and fixed to a foam platform using non-adhesive tape and pins. A length of MA silk was manually drawn from the spinnerets, adhered to a mechanical spool with masking tape and reeled at 1 m min^−1^ for 1 h to ensure the store of MA silk was collected for both the pre- and post-treatment samples from the MA spinnerets of every spider. We used a dissecting microscope to observe the spinnerets to ensure a single thread of MA silk was consistently drawn and there was no intervention by other spinnerets.

Six 25 mm sections of MA silk from each spider (total samples = 6×21 individuals×3 species = 378) were mounted onto 20×20 mm cardboard frames, containing double-sided adhesive tape around a 5 mm border. A second cardboard frame with double-sided adhesive tape around its border was placed on top of the original, and the frames were stuck together, securing the silk within [32]. The frames containing silk were taped to a microscope slide and examined and photographed under a polarized light microscope (Olympus BX50, Tokyo) connected to a UC-series Nikon digital camera. The diameter of each thread was determined from the photographs using the program Image J (NIH, Bethesda MD, USA) to account for it in mechanical tests. The remaining silk extracted from each individual was weighed to the nearest 0.01 mg on an electronic balance and placed into 10 µl tubes (Eppendorf, Hamburg, Germany). High performance liquid chromatography [2] was then performed to identify the composition of all amino acids in the silks using a Pico-Tag Amino Acid Column (Waters Milford CA, USA) after submergence in 6 mol l^−1^ hexafluoro-isopropanol and hydrolysis in 6 mol l^−1^ HCl for 24 h [4].

Mechanical tensile tests were done on the frame-mounted silks using a UTM Micro Bionix tensile tester (MTS Systems Corporation, Oakridge TN, USA) within 48 h of collection at the Centre for Measurement Standards, Industrial Technology Research Institute, Hsinchu, Taiwan, under controlled temperature (∼20°C) and humidity (∼30%). The silks were stretched at a strain rate of 1% of the gage length per second until rupture. The load resolution varied from 2–10 µN depending on the diameter of the silk tested. Engineering stress (σ) and strain (ε) were calculated [51] and stress-strain curves were plotted using TestWorks 4.0 (MTS Systems Corporation, Eden Prairie MN, USA). Using the stress-strain curves, we calculated the following parameters: (1) ultimate strength; or stress at rupture, (2) extensibility; or strain at rupture, (3) toughness; or area under the stress strain curve and (4) Young's modulus (stiffness); or the slope of the curve during the elastic phase.

### Analyses

As the percentage composition of any given amino acid in a protein-based material is dependent on the composition of other amino acids [7] we treated the percent composition of each amino acid as non-independent. We statistically compared the change in mean (μ_post_-μ_pre_) percent compositions of silk glutamine, serine, proline, glycine and alanine, as these comprise >95% of the amino acids in spider MA silk [52], [53], by a paired (within individuals) full-effects multivariate analysis of variance (MANOVA). The within-individual paired comparisons were done to account for the possibility that some stored silk in the pre-treatment silks rendered non-uniformity across individuals. The independent variables included in the MANOVA were: (1) species (*A. aetherea*, *C. moluccensis* and *L. blanda*) and (2) feeding treatment (HP, LP or NP). To account for the possibility that differences in common ancestry among the species biases the between-species comparisons [54], the analyses were made by contrasting the phylogenetic branch lengths, determined in arbitrary units from a recent spider phylogeny [43], using pairwise comparisons [54], [55]. We performed a further full-effects MANOVA, using independent contrasts of phylogenetic branch lengths [55], to compare any changes in mechanical properties (ultimate strength, toughness, extensibility and Young's modulus) in MA silk across treatments and species. We performed Newman-Keuls Critical Range *post-hoc* tests where significant μ_post_-μ_pre_ differences between treatments or species were identified to determine the treatment(s) responsible for altering amino acid composition or mechanical properties. Amino acid compositions were measured as a percentage of total amino acids present and thread extensibility was measured as the percent extension beyond the original gage length. These data were accordingly arcsine transformed to fit the MANOVA assumptions.

We used multiple linear regression analysis to determine the relationships between protein intake (scored as 56%, 25% and 0% for the HP, LP and NP treatments respectively), the phylogenetic branch lengths between species, the compositions of the five major amino acids (glutamine, serine, proline, glycine, and alanine), silk mechanical properties (ultimate strength, extensibility, toughness and Young's modulus) and thread diameter. A correlation matrix was developed from the subsequent regression model [56] to ascertain the proximal and distal affects of protein intake and phylogenetic branch lengths on silk amino acid composition and mechanical property variations. The data for the three species was combined for this analysis.

## Results

### Influences on amino acid compositions

Significant variations in the amino acid compositions were found between pre- and post-feeding MA silks and these variations differed between species and treatments when inter-specific phylogenetic relationships and species×treatment interactions were accounted for (Table 1; Table 2).

**Table 1:** Results of a multi-factorial analysis of variance (MANOVA) to compare the change in mean (μ_post_-μ_pre_) percent composition of five amino acids: glutamine, serine, proline, glycine, and alanine.

	Wilk's *λ*	Rao's *R*	*df*	*p*
Species	0.003	96.089	10,64	<0.0001
Treatment	0.090	14.867	10,64	<0.001
Species×treatment	0.059	7.204	20,107	<0.001

The independent variables are: (1) species (*A. aetherea*, *C. moluccensis* and *L. blanda*) and (2) treatment (HP, LP or NP).

**Table 2:** Mean (±SE) pre (μ_pre_) and post (μ_post_) treatment amino acid compositions of *Argiope aetherea* (a), *Cyrtophora moluccensis* (b) and *Leucauge blanda* (c) MA silks, showing the results of a paired MANOVA (*F*-scores) and Newman-Keuls (N-K) *post-hoc* tests comparing μ_pre_–μ_post_ compositions between treatments.

Amino acid		(1)	(2)	(3)	*F*_2,22_	*p*	N-K test:
		HP	LP	NP			
a) *Argiope aetherea*							
GLU	μ_pre_	6.68±1.99	7.26±1.81	8.14±0.69	31.68	<0.0001	1 = 2≠3
	μ_post_	10.30±0.13	10.67±0.16	5.80±0.23			
SER	μ_pre_	4.42±0.41	4.84±0.36	3.89±0.13	2.93	0.043	1 = 2 = 3
	μ_post_	5.17±0.19	4.23±0.27	4.89±0.21			
PRO	μ_pre_	11.00±0.36	11.57±0.33	10.7±0.51	54.98	<0.0001	1≠2≠3
	μ_post_	12.22±0.78	11.64±0.16	7.18±0.79			
GLY	μ_pre_	35.02±2.79	36.28±1.03	36.39±3.13	4.32	0.01	1 = 2≠3
	μ_post_	37.31±0.25	38.09±0.52	36.38±0.45			
ALA	μ_pre_	22.18±4.52	22.06±2.09	21.69±1.65	13.28	<0.001	1 = 2≠3
	μ_post_	17.64±0.63	17.89±0.50	23.65±0.56			
b) *Cyrtophora moluccensis*							
GLU	μ_pre_	5.61±0.28	4.21±0.76	6.06±0.61	3.14	0.03	1 = 2≠3
	μ_post_	5.44±0.57	5.18±0.61	2.76±0.81			
SER	μ_pre_	3.68±0.16	3.78±0.31	4.21±0.24	2.65	0.06	−−
	μ_post_	4.92±0.57	3.31±0.34	3.45±0.79			
PRO	μ_pre_	2.75±0.28	2.26±0.46	2.98±0.38	2.61	0.07	−−
	μ_post_	2.54±0.36	3.06±0.89	2.23±0.54			
GLY	μ_pre_	32.09±0.67	34.11±1.71	32.11±0.74	0.62	0.27	−−
	μ_post_	31.34±1.23	31.94±1.94	34.32±1.31			
ALA	μ_pre_	36.35±1.67	35.83±1.41	34.60±1.09	0.44	0.78	−−
	μ_post_	35.39±1.43	35.82±0.51	33.93±1.61			
c) *Leucauge blanda*							
GLU	μ_pre_	8.10±0.21	7.78±0.46	7.19±0.46	2.34	0.08	−−
	μ_post_	7.31±0.54	6.07±0.59	6.22±0.76			
SER	μ_pre_	5.77±0.12	6.46±0.56	5.62±0.61	2.77	0.05	1≠2 = 3
	μ_post_	7.77±0.68	4.53±0.79	4.04±0.34			
PRO	μ_pre_	12.16±0.13	10.73±0.54	10.29±0.93	33.53	<0.0001	1≠2≠3
	μ_post_	10.54±0.67	6.07±1.54	2.12±0.41			
GLY	μ_pre_	37.36±0.67	36.42±0.91	34.32±1.33	0.75	0.56	−−
	μ_post_	34.16±1.45	31.28±1.75	33.92±1.74			
ALA	μ_pre_	13.55±0.75	13.34±1.40	13.81±1.41	11.91	<0.001	1≠2≠3
	μ_post_	17.05±2.96	24.36±5.80	34.72±1.38			

Treatments = high protein (HP), low protein (LP) and no protein (NP) food for 10 days.

For *Argiope aetherea* the composition of glutamine, serine, proline, glycine, and alanine in their MA silk all differed significantly pre- compared to post-treatment (Newman-Keuls tests; *p*<0.05; Table 2a). Glutamine and proline compositions increased when feeding on the HP and LP solutions but decreased when feeding on the NP solution. Glycine composition increased when feeding on the HP and LP solutions but did not change when feeding on the NP solution. Alanine decreased when feeding on the HP and LP solutions but increased when feeding on the NP solution. These results suggest there was an increase in the number of poly-A motifs and a reduction in –GPG motifs, which is consistent with a shift toward greater MaSp1 expression under the MaSp model.

In contrast to *A. aetherea*, only the glutamine composition of *Cyrtophora moluccensis* MA silk significantly varied pre- compared to post-treatment (Newman-Keuls tests; *p*<0.05; Table 2b), decreasing when feeding on the NP treatment while remaining relatively unchanged when feeding on the HP and LP treatments. Serine and proline composition decreased in *C. moluccensis* MA silk post-treatment, but this decrease was not significantly different whether fed the HP, LP or NP solutions.

The pre- compared to post-treatment serine, proline and alanine composition of *Leucauge blanda* MA silk differed across all treatments (Newman-Keuls tests; *p*<0.05; Table 2c). Its serine composition increased on the HP treatment but decreased on the LP and NP treatments. Its proline composition decreased to different degrees in all treatments, decreasing most austerely (from ∼10 to ∼2%) when fed the NP treatment. In contrast, its alanine content increased to different degrees on all treatments, increasing most austerely (from ∼14 to ∼45%) when fed the NP treatment. Such a result suggests a sizeable increase in the number of poly-A motifs and a reduction in –GPG motifs, consistent with a shift toward greater MaSp1 expression.

### Influences on mechanical properties

While we found significant variations in mechanical properties between the pre- and post-experimental MA silks, the variations differed only between species and species×treatment interactions (Table 3). The mechanical properties that varied included: (*i*) extensibility, which varied in all three species by decreasing sequentially between the HP, LP and NP treatments (Table 4), (*ii*) ultimate strength, which was significantly lower in *L. blanda* MA silks from the HP treatment compared to those on the LP or NP treatments (Table 4c), and (*iii*) Young's modulus, which was significantly lower in *L. blanda* MA silks from the NP and LP treatments compared to those on the HP treatment (Table 4c).

**Table 3:** Results of a multi-factorial analysis of variance (MANOVA) to compare the change in mean (μ_post_-μ_pre_) mechanical properties; ultimate strength, toughness, extensibility and Young's modulus.

	Wilk's *λ*	Rao's *R*	*df*	*p*
Species	0.123	15.288	8,66	<0.001
Treatment	0.668	1.845	8,66	0.084
Species×treatment	0.359	2.529	16,101	0.002

The independent variables are (1) species (*A. aetherea*, *C. moluccensis* and *L. blanda*) and (2) treatment (HP, LP or NP).

**Table 4:** Mean (± SE) pre- (μ_pre_) and post- (μ_post_) treatment mechanical properties: ultimate strength (MPa), extensibility (%), toughness (MJ/m^3^), Young's modulus (GPa), and thread diameter (µm) of *Argiope aetherea* (*a*), *Cyrtophora moluccensis* (*b*) and *Leucauge blanda* (*c*) MA silks, showing the results of a paired MANOVA (*F*-scores) and Newman-Keuls (N-K) *post-hoc* tests comparing μ_pre_–μ_post_ compositions between treatments.

Mechanicalparameters		(1)	(2)	(3)	*F*_2,22_	*p*	N-K test:
		HP	LP	NP			
(*a*) *Argiope aetherea*							
Ultimate strength	μ_pre_	640.19±55.61	592.68±52.75	589.62±86.15	0.65	0.557	−−
	μ_post_	877.77±75.64	754.59±59.61	699.86±83.24			
Extensibility	μ_pre_	36.12±0.12	35.82±0.45	36.43±0.54	9.08	0.01	1≠2 = 3
	μ_post_	41.35±0.25	26.26±0.35	21.23±0.12			
Toughness	μ_pre_	184.40±27.27	198.03±33.67	205.37±32.87	2.81	0.098	−−
	μ_post_	227.07±25.94	205.05±25.28	168.90±31.59			
Young's modulus	μ_pre_	8.71±1.32	8.21±0.46	8.26±1.13	0.48	0.625	−−
	μ_post_	6.25±0.79	6.44±0.67	6.66±0.47			
Thread diameter	μ_pre_	3.82±0.13	4.09±0.43	3.96±0.31	1.04	0.381	−−
	μ_post_	3.85±0.65	3.68±0.21	4.05±0.73			
(*b*) *Cyrtophora moluccensis*							
Ultimate strength	μ_pre_	809.99±22.64	759.11±34.98	836.35±55.57	0.19	0.826	−−
	μ_post_	740.44±43.43	704.24±53.61	720.21±68.63			
Extensibility	μ_pre_	23.42±0.61	24.22±0.35	27.26±0.34	12.46	0.001	1≠2≠3
	μ_post_	43.88±0.09	24.21±0.27	20.08±0.55			
Toughness	μ_pre_	231.87±55.81	222.32±47.88	244.82±33.73	1.36	0.291	−−
	μ_post_	195.68±61.89	240.65±53.72	241.74±14.63			
Young's modulus	μ_pre_	10.15±2.06	9.12±0.43	9.78±0.66	1.13	0.352	−−
	μ_post_	10.44±0.83	9.01±0.37	8.37±1.64			
Thread diameter	μ_pre_	3.91±0.74	3.54±0.30	3.41±1.01	2.52	0.121	−−
	μ_post_	3.57±0.47	3.71±1.17	3.25±0.16			
(*c*) *Leucauge blanda*							
Ultimate strength	μ_pre_	644.69±57.92	641.28±53.01	685.79±69.33	10.42	0.002	1≠2 = 3
	μ_post_	499.19±53.50	571.61±16.75	637.35±66.14			
Extensibility	μ_pre_	21.74±0.44	22.13±0.34	24.06±0.75	10.07	0.003	1≠2 = 3
	μ_post_	29.01±0.37	16.21±0.33	15.61±0.27			
Toughness	μ_pre_	92.22±13.81	118.45±34.19	105.42±28.95	0.16	0.848	−−
	μ_post_	106.86±3.77	103.90±17.01	85.63±8.21			
Young's modulus	μ_pre_	8.33±0.47	9.03±2.73	8.56±0.45	12.27	0.001	1≠2 = 3
	μ_post_	8.78±0.73	7.22±1.04	6.34±1.48			
Thread diameter	μ_pre_	2.41±0.25	2.33±0.34	2.36±0.34	1.08	0.369	−−
	μ_post_	1.98±0.87	2.44±0.54	2.11±0.17			

Treatments = high protein (HP), low protein (LP) and no protein (NP) food over 10 days.

### Influence of phylogeny and protein intake on silk properties

Multiple regression of all three species data combined found that extensibility, Young's modulus and thread diameter were influenced by a combination of variations in amino acid compositions, phylogenetic branch lengths (i.e. “phylogenetic inertia” [54], [57]) between species, and protein intake (Table 5). The composition of proline and glycine was positively associated with extensibility and negatively associated with Young's modulus, the composition of serine was positively associated with ultimate strength while glycine and alanine compositions were negatively associated. Protein intake was positively associated with glutamine and proline compositions, and negatively associated with alanine compositions (Table 6). According to the correlation coefficients that we derived (Table 6), the influences of protein intake and amino acid composition on mechanical properties were generally weaker than phylogenetic influences.

**Table 5:** Regression analysis between MA silk mechanical properties; ultimate strength, extensibility, toughness, Young's modulus and thread length, and the compositions of the amino acids glutamine, serine, proline, glycine, and alanine, treatment (HP, LP or NP entered as 56%, 25% and 0% protein respectively), and the phylogenetic branch lengths (in arbitrary units derived from [43]).

	*β*	SE	*t*_38_	*p*
Intercept	–	–	2.381	0.021
Ultimate strength	0.203	0.186	1.109	0.281
Extensibility	0.515	0.205	2.256	0.016
Toughness	0.106	0.188	0.566	0.574
Young's modulus	−0.305	0.143	−2.130	0.036
Thread diameter	−5.91	0.244	−2.141	0.021

Data for the three species is combined.

**Table 6:** The correlation matrix derived from a multiple regression between MA silk mechanical properties; ultimate strength (US), extensibility, toughness, Young's modulus (YM) and thread diameter (TD), and the compositions of the amino acids glutamine (GLU), serine (SER), proline (PRO), glycine (GLY), and alanine (ALA), treatment (HP, LP or NP entered as 56%, 25% and 0% protein respectively), and phylogenetic branch lengths (BL; in arbitrary units derived from [43]).

	Amino acids						
	GLU	SER	PRO	GLY	ALA	BL	Treatment
US	0.02	**0.60**	0.09	**−0.57**	**−0.60**	**−0.62**	0.03
Extensibility	0.25	−0.21	**0.43**	**0.45**	0.28	**−0.74**	0.19
Toughness	0.25	−0.21	0.04	0.40	0.35	**−0.67**	−0.09
YM	−0.27	0.07	**−0.56**	**−0.43**	0.40	0.17	0.08
TD	−0.13	**−0.39**	−0.07	0.36	0.35	**−0.70**	−0.32
BL	0.08	**0.60**	0.09	**−0.57**	**0.60**	1.00	0.00
Treatment	**0.33**	0.01	**0.31**	0.09	**−0.30**	0.00	1.00

Bold text indicates significant correlations (*p*<0.05).

### Discussion

Here we showed, accounting for phylogenetic relationships, that the chemical and physical properties of a secreted proteinaceous material, the MA silks of the orb web spiders *Argiope aetherea*, *Cyrtophora moluccensis* and *Leucauge blanda*, vary with the concentration of protein ingested. Moreover, our analyses revealed that while silk amino acid composition variations were proximately influenced by the concentration of protein that spiders take up the variations in the mechanical properties of their MA silk were influenced principally by phylogeny, with protein intake only influencing variations in mechanical properties via its influences on amino acid composition. The protein concentrations of the solutions used herein reflect the extremes of protein concentrations found naturally in insects [1], [47], [48]. Thus our study demonstrates, albeit making the untested assumption that spiders can extract and metabolize albumin protein and energy in precisely the same way as insect-derived protein and energy, the kind of metabolic and physiological adjustments that spiders make in order to modify their silks in response to changes in their nutritive environment.

Variations in the amino acid composition of *A. aetherea* (a spider with MA silks that are predicted to predominantly contain MaSp2) MA silk showed more similarities to *L. blanda*'s (who is distantly related to *A. aetherea* but has MA silks also predicted to contain predominantly MaSp2) MA silk than to the more closely related *C. moluccensis* (who is predicted to have predominantly MaSp1 MA silk). Therefore it appears that silk type, i.e. predominance of MaSp1 or MaSp2, and the energetic costs of synthesizing each silk type, is most likely the driver of the shifts in MA silk amino acid compositions in response to the intake of different protein concentrations. Similar variations in silk amino acid compositions have been reported for *Nephila clavipes* in response to starvation [8], [25]. Our results thus support the proposition that protein uptake via food or web re-ingestion is primarily relied upon to supply the amino acids in silk that are energetically expensive to synthesize, e.g. glutamine and proline. Much of the amino acids consumed nonetheless appear to be broken down and re-synthesized before incorporation into silk. For instance, the HP and LP solutions had a high concentration of lysine, cytosine and asparagine but these did not seem to be incorporated into the silks as, while not statistically analyzed, their compositions in all MA silks remained relatively low (0–2%). On the other hand, despite being absent from the NP solution, alanine composition increased in both *A. aetherea* and *L. blanda* MA silks when fed that treatment.

While the MA silk amino acid compositional variations across the NP, LP and HP treatments in our study exhibited some similarities with previous starvation experiments [8], [25], there are some important differences. For instance, we found that glutamine was reduced in *C. moluccensis* silks when feeding on the NP solution without any concurrent reduction in proline. Likewise, the reduction in glutamine and proline in *A. aetherea* was not accompanied by similar decreases in serine. According to the MaSp model developed for *N. clavipes*, if variations in the MaSp1:2 ratio were responsible for the shifts in silk expression, then proline (which is exclusively found in MaSp2), glutamine and serine (which are more prominent in MaSp2) should co-vary. Proline and serine were found to be lower, albeit insignificantly, in *C. moluccensis* MA silks when feeding on the NP solutions, so it is possible that MaSp2 down-regulation occurs when protein intake ceases. Nonetheless in a previous experiment with *Nephila pilipes* fed different diets similar MA silk compositional variations for glutamine and serine were found without concomitant variations in proline composition [32]. These and other published discrepancies to the MaSp model, e.g. the compositional and mechanical responses of the MA silks of *Cyclosa mulmeinensis* and *C. ginnaga* when exposed to wind [58], allude to the possibility that the model is not able to predict MA silk amino acid compositional and mechanical property variations across all spiders. We suggest that more silks need to be examined at a molecular level to establish species-specific models.

Researchers have recently found various MA silk gene duplicates among different spiders [18], [59]–[61], so it is plausible that there are more than two spidroin genes in any of the three species that we used. We performed liquid chromatography to derive across treatment amino acid compositions for the MA silks of the three species used. While this is a widely used technique and adequate for making standardized across and between treatment intra- and inter-specific comparisons [2], [8], [32], any comparison with other studies should consider that other methods with different levels of precision may have been used to derive amino acid composition. Nuclear magnetic resonance (NMR) and other spectroscopic methods are becoming more widely used in silk research [14], [19], [25], [62] as these methods have lower amino acid compositional variability associated with their analyses compared to traditional methods [14], [62], [63]. We therefore cannot rule out MaSp expression as a means by which the MA silks of the spiders examined here vary with protein intake until adequate comparable NMR studies are done.

Our analysis suggests that the concentration of protein taken up proximally induces variations in silk extensibility and stiffness via its influence on glutamine, proline and alanine compositions across the three species examined. In *N. clavipes*, alanine is predicted to be involved in the formation of crystalline β-sheets while proline disrupts crystallite growth [12], [19], [22]. Moreover, β–sheet formation is associated with greater ultimate strength and reduced extensibility in spider silk [20], [21]. Accordingly, the reduced proline and enhanced alanine should result in less extensible, stronger silks. We found that extensibility was reduced when silk proline composition was low but a concomitant increase in ultimate strength with an increase in alanine composition was not found. Our results thus contradict expectations if MaSp2 down-regulation was the principal mechanism driving mechanical property variations. Factors other than proline and alanine composition, such as ionic, hydration, pH and temperature variations within the silk gland and/or haemolymph can influence the size and density of crystals and the formation of strength-enhancing β–sheets in MA silk [11], [36]–[38], [64]. Any of these factors may have been altered as the spiders experience metabolic stress and, accordingly, may have affected the silk mechanics.

To summarize, we demonstrate that the level of protein intake influences spider silk expression in *A. aetherea*, *C. moluccensis* and *L. blanda*. All of these species reduced their glutamine, proline and/or serine compositions in response to low/no protein diets but they did so to different extents. Our results support the premise that amino acids derived from citric acid cycle metabolites, e.g. glutamine and proline, are associated with a higher sacrifice of metabolic energy [4] so protein intake via food or web re-ingestion is largely relied upon for their incorporation into silk [8], [9]. Most orb web spiders have MA silks high in MaSp2 [9]. As MaSp2, owing to its higher glutamine, proline and serine, is energetically expensive to synthesize it may be down-regulated by orb web spiders when protein intake is limited [8] and our results partially support this. Nonetheless, at least one of the spiders tested, *C. moluccensis*, did not exhibit significant reductions in proline composition when on the low or no protein treatment. The mechanical properties of the silks varied with protein intake but these variations were contradictory to the expectations of MaSp2 down-regulation. Our analyses suggested they were more influenced by phylogenetic intertia between related species. As we do not know the genetic inputs into the MA silks of the spiders that we used we cannot speculate as to whether or not spidroin orthologues or other proteins are responsible for the contradictions to the MaSp model. We however expect spinning conditions to differ for each spider and for these to influence silk mechanical properties independent of MaSp expression [11], [15], [25], [36]–[38].

One implication of our study is that since different spiders produce silks of different MaSp1 and MaSp2 composition (e.g. *A. aetherea* and *L. blanda* probably produce silks richer in MaSp2 than *C. moluccensis*) different spiders probably adjust their silk properties in different ways in response to protein limitation just as variable responses to a varying nutrient environments has been shown for spider life history traits [40], [47]–[50], [65]–[67]. Another implication is that many orb web spiders that are predicted, based on proline composition, to have MaSp1 predominant silk also build either three dimensional webs or two dimensional orb webs with three dimensional barrier structures, e.g. *Nephila clavipes*, *Latrodectus hesperis* and *Cyrtophora* spp. [8], [18], [20]–[23]. These three-dimensional webs alleviate the requirement to produce highly extensible silks to capture insects in full flight [68]. Accordingly, if three-dimensional web building spiders can utilize MaSp1 predominant silks to capture prey, the requirement of extracting the majority of their protein for silk synthesis from food or web re-ingestion may be alleviated. More data on the chemical and physical properties and MA silk gene sequences for two-dimensional and three-dimensional web-building spiders are needed however to corroborate our conjecture.
